# An Unusual Case of Primary Sjögren’s Syndrome and Lymphangioleiomyomatosis Diagnosed From Refractory Cough

**DOI:** 10.7759/cureus.89956

**Published:** 2025-08-13

**Authors:** Ruo-Nan Yan, Yi Zhang, Shu-guang Yang, Xue-qing Yu

**Affiliations:** 1 Department of Respiratory Medicine, The First Affiliated Hospital of Henan University of Chinese Medicine, Zhengzhou, CHN; 2 The First College for Clinical Medicine, Henan University of Chinese Medicine, Zhengzhou, CHN

**Keywords:** case report, lymphangioleiomyomatosis, multidisciplinary diagnosis, primary sjögren’s syndrome, refractory cough

## Abstract

Primary Sjögren’s syndrome (pSS) is a chronic autoimmune disorder that can involve the lungs, leading to interstitial lung disease (ILD) and lymphoproliferative disorders. Lymphangioleiomyomatosis (LAM) is a rare neoplastic disorder in ILD. Co-occurrence of these diseases is exceptionally rare. We report the case of a 47-year-old woman who presented with a persistent cough, initially misdiagnosed as a nonspecific respiratory condition. Empirical therapy proved ineffective. As her cough worsened and was accompanied by xerostomia and xerophthalmia, a comprehensive examination prompted a diagnosis of pSS. Although glucocorticoids and immunomodulatory therapy alleviated her sicca symptoms, the cough persisted. High-resolution computed tomography revealed cystic cavities, which were initially mistaken for bullous emphysema. The refractory cough prompted further investigation, and lung biopsy and elevated serum vascular endothelial growth factor-D confirmed LAM. Sirolimus significantly ameliorated the cough severity, while corticosteroid combined with immunosuppressive agents was continued for pSS management. Follow-up found that the patient’s symptoms remained stable. This rare case underscores the diagnostic challenges of overlapping rare diseases and highlights the critical role of multidisciplinary assessment, pulmonary imaging, and histopathological examination in achieving an accurate diagnosis.

## Introduction

Primary Sjögren’s syndrome (pSS) is a multisystem autoimmune disease, characterized by hypofunction of salivary and lacrimal glands or systemic multi-organ manifestations [1,2]. The prevalence rate is estimated between 0.02% and 2.7% [3]. Approximately 10-20% of pSS cases demonstrate pulmonary involvement with lower quality of life and higher mortality [4].

Lymphangioleiomyomatosis (LAM) is a rare, progressive cystic lung disease mainly affecting women of childbearing age, characterized by dyspnea, cough, and diffuse pulmonary cystic changes [5]. Currently, the coexistence of pSS and LAM is rare and has not been reported, posing significant diagnostic and therapeutic challenges. This case report documents the first known instance of pSS and LAM coexistence, emphasizing the need for heightened clinical suspicion and multidisciplinary evaluation.

## Case presentation

History of present illness

The patient, a 47-year-old woman, had an intermittent cough for more than four years, which had worsened over the past two weeks. In the winter of 2020, the patient developed an intermittent dry cough, exacerbated by cold air or cooking fumes, without nasal congestion, rhinorrhea, dry or sore throat, chest tightness, or dyspnea. The patient took cough suppressants orally, but the symptoms did not improve. Recurrent winter exacerbations persisted, lasting from October to March annually, with increasing severity. The patient visited local clinics and hospitals multiple times for empirical antitussives and antibiotics (details unavailable), without definitive etiological identification, but relief was minimal. By October 2022, the patient developed additional symptoms, including intermittent xerostomia and xerophthalmia, with occasional fragmentary tooth loss, prompting evaluation at the First Affiliated Hospital of Zhengzhou University.

When the patient was admitted to the First Affiliated Hospital of Zhengzhou University, apart from the above symptoms, no accompanying fever, fatigue, or Raynaud’s phenomenon was observed. Initial assessment revealed bilateral pulmonary bullae and emphysematous changes on lung computed tomography (CT). Serological testing showed markedly elevated anti-SSA and anti-Ro-52 autoantibodies. A lip gland biopsy indicated mild lymphocytic infiltration, insufficient for a definitive diagnosis of pSS according to the 2016 American College of Rheumatology/European League Against Rheumatism (ACR/EULAR) classification criteria [1]. However, clinical and serological findings supported a pSS diagnosis. According to the patient’s lung CT, it was suspected that the cough might be attributed to traction bronchiectasis secondary to pulmonary involvement of pSS, so treatment still targeted pSS. Treatment included immunomodulators, anti-inflammatory agents, and symptomatic relief for sicca symptoms. After discharge, the patient took medication regularly, but the intermittent cough persisted for the next four years.

In October 2024, the patient’s syndromes worsened, accompanied by dry skin and grade II elbow joint tenderness (without swelling or pain). Accordingly, she was re-evaluated at the Rheumatology Department of the First Affiliated Hospital of Henan University of Chinese Medicine. Lung CT revealed multiple cystic lucencies in both lungs. While the original immunomodulatory and anti-inflammatory therapy was maintained, rituximab was added, yet cough control remained suboptimal. After consulting with a respiratory specialist, the suspicion of LAM was raised, based on CT findings and pSS history. The patient received immunomodulatory therapy combined with traditional Chinese medicine, resulting in a modest improvement in cough symptoms. Because management was empiric without a definitive diagnosis, she was advised to undergo a lung biopsy for definitive confirmation. Histopathology revealed spindle cell proliferation with immunohistochemical positivity for human melanoma black-45 (HMB-45), progesterone receptor (PR), estrogen receptor (ER), and smooth muscle actin (SMA), confirming LAM. Elevated serum vascular endothelial growth factor-D (VEGF-D) further supported the diagnosis. Sirolimus was initiated, reducing cough severity within one week. In the following days, the patient took medication orally on a regular basis.

Two weeks before the latest admission (March 2025), a low-grade fever (peak temperature 37.3 ℃), sore throat, rhinorrhea, nasal congestion, and cough recurred after a cold. The patient took amoxicillin orally, which resolved the fever and nasal symptoms, but the cough persisted. On admission, the patient coughed severely, and physical examination revealed pharyngeal hyperemia without fever, dyspnea, nasal congestion, or gastroesophageal symptoms (acid reflux or heartburn). Nutritional intake and sleep patterns remained unimpaired, with no significant weight fluctuations in the preceding weeks.

**Figure 1:**
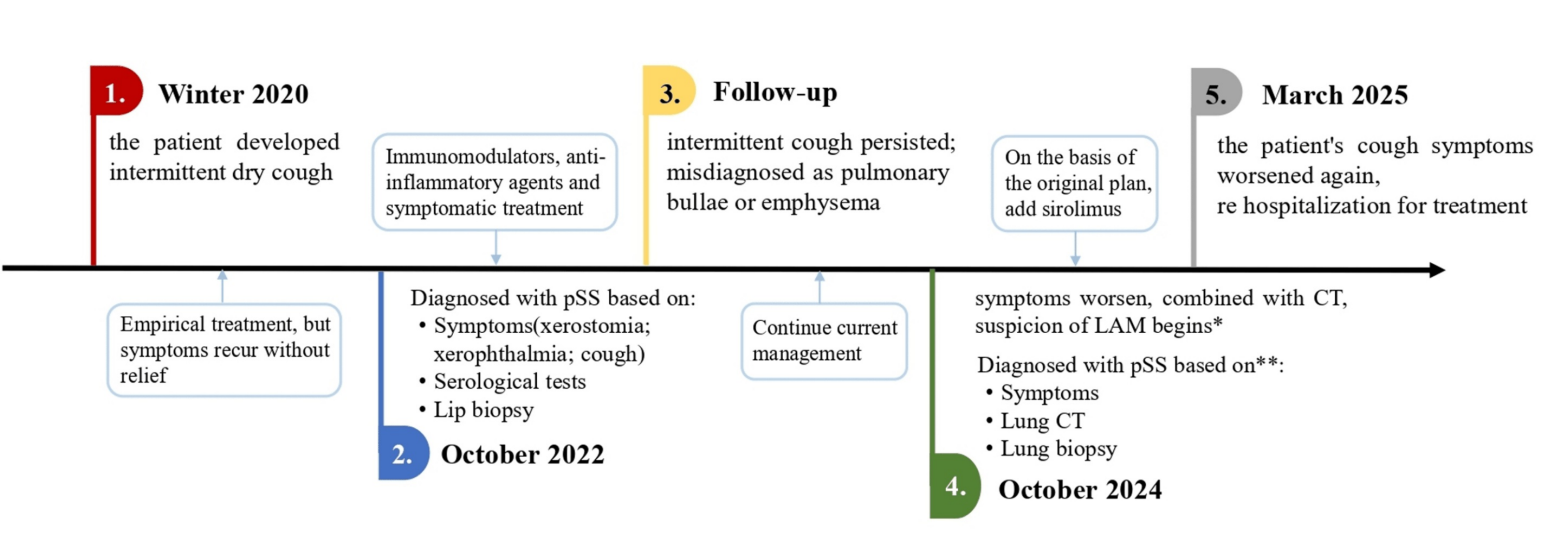
Timeline of patient diagnosis and treatment *In October 2024, the patient was initially evaluated at the First Affiliated Hospital of Henan University of Chinese Medicine, where lung CT findings raised clinical suspicion of LAM. ** Subsequent evaluation at the First Affiliated Hospital of Guangzhou Medical University established a definitive diagnosis of LAM through histopathological confirmation via lung biopsy and ancillary diagnostic testing. LAM: lymphangioleiomyomatosis; pSS: primary Sjögren’s syndrome

To better visualize the disease progression, we chronologically delineate key milestones, including symptom onset, pSS diagnosis, LAM identification, and treatment initiation (Figure 1).

History of past illness and family history

The patient was diagnosed with pSS in October 2022 and LAM in October 2024. There was no history of other past illnesses. There was no personal or family history.

Physical examination upon admission

On admission in March 2025, the body temperature was 36.3℃, pulse 80 beats/min, respiratory rate 20 breaths/min, and blood pressure 118/75 mmHg. The patient's body height was 165 cm, and weight was 65 kg. During hospitalization, the body temperature, pulse, respiratory rate, and blood pressure remained normal. Alertness and cooperation were good. No rashes or bleeding spots were observed throughout the body. The superficial lymph nodes were not palpable anywhere on the whole body. The conjunctivae were not hyperemic. The lips were not cyanotic; the oral mucosa was smooth; pharyngeal hyperemia was obvious; the tonsils were good; and no purulent secretions were observed. The neck was soft and without resistance. On auscultation, heart and breathing sounds were normal. Abdominal examination was normal, and nervous system examination was normal.

Laboratory examinations

**Figure 2:**
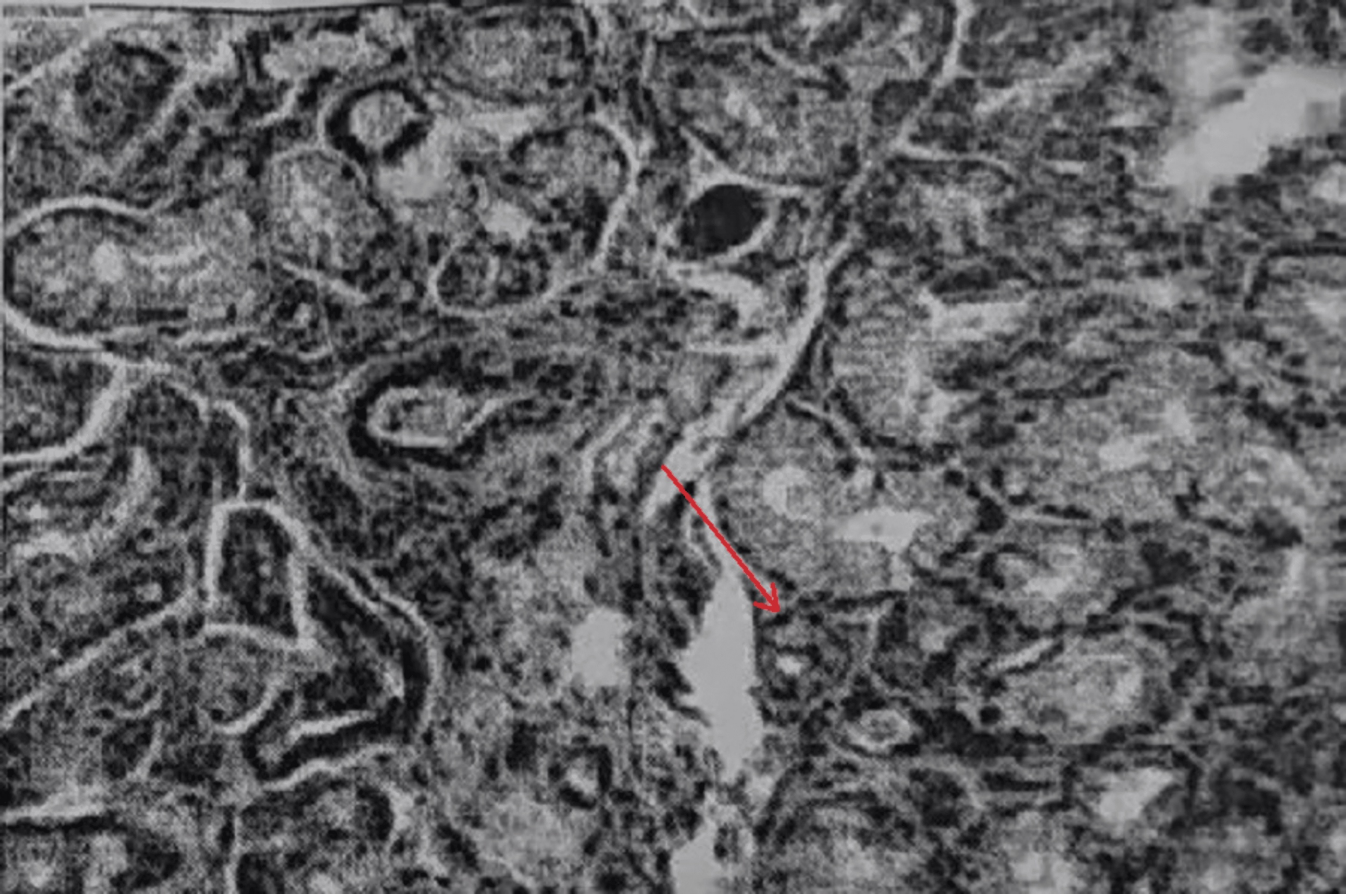
Histpathological image of lip gland tissue. The histopathological image of the lip gland tissue shows a small amount of lymphocyte and plasma cell infiltrates.

**Figure 3:**
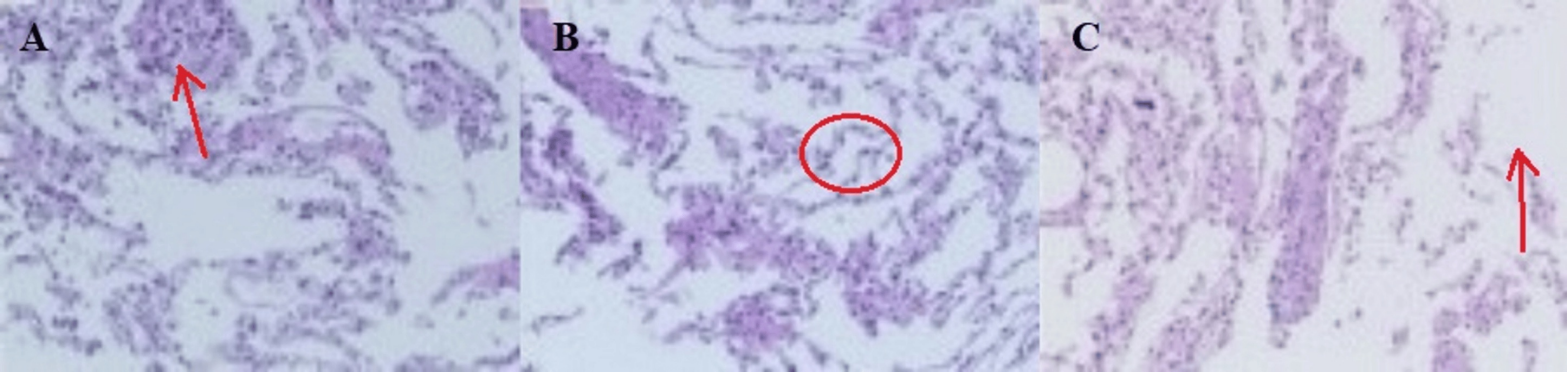
Pathological images of lung tissue (A) Scattered tissue cells are visible in the lung parenchyma; (B) lymphocytic infiltration can be observed within the alveolar spaces; (C) slight proliferation of spindle shaped cells can be seen locally, with the formation of pulmonary bullae.

At the First Affiliated Hospital of Zhengzhou University (October 2022), the results of the lip gland biopsy showed that the salivary glands had a lobular structure. A small number of lymphocytes and plasma cells were infiltrated in the local stroma, but the number of lymphocytes was relatively low. The lesion did not meet the diagnostic criteria for pSS. It was suggested that the diagnosis be based on the patient’s clinical manifestations (Figure 2). In October 2024, the patient underwent bronchoscopy at the First Affiliated Hospital of Guangzhou Medical University. Pathological examination was performed on some lung tissues, and a few histiocytes could be seen in the alveolar lumens. The interstitium was slightly widened, with lymphocytic infiltration. Some interstitia were broken, and bullae were formed. Focally, a few proliferating spindle cells could be seen, with no obvious atypia. Immunohistochemical results included the following: HMB-45 (individual +), PR (small amount +), ER (+), and SMA (+). Combined with the immunohistochemical findings, the histological changes were suggestive of LAM (Figure 3).

Notable findings from the laboratory tests were persistently elevated anti-SSA and anti-Ro-52 autoantibodies and elevated VEGF-D (Table 1). Pulmonary function tests showed mild diffusion impairment (Table 2).

**Table 1:** Laboratory tests C3: complement component 3; C4: complement component 4; IgG: immunoglobulin G; IgA: immunoglobulin A; IgM: immunoglobulin M; ANA: antinuclear antibody; dsDNA: anti-double-stranded DNA antibody; Ro52: anti-Ro52 antibody; SSA: anti-SSA antibody; SSB: anti-SSB antibody; VEGF-D: vascular endothelial growth factor-D; NA: not available. * nuclear particles + homogeneous type. ^#^ Negative result, no exact value.

Item	Result (2022.10)	Result (2024.10)	Result (2025.03)	Reference Range
Immunoglobulin and complement				
C3 (g/L)	2.10	1.47	NA	0.9–1.8
C4 (g/L)	0.22	0.52	NA	0.1–0.4
IgG (g/L)	13.4	10.30	NA	7–16
IgA (g/L)	2.0	1.28	NA	0.7–4
IgM (g/L)	0.8	0.46	NA	0.4–2.3
Autoantibody				
ANA (IgG)	1:320 (+)^*^	(-) ^#^	(-) ^#^	NA
dsDNA (IgG)(IU/ml)	27.80	8.30	3.80	0–26.9
Ro52 (RU/ml)	＞400	274.32	247.70	0–20
SSA (RU/ml)	＞400	＞200	＞200	0–20
SSB (RU/ml)	3.98	10.70	10.70	0–20
VEGF-D (pg/ml)	NA	837.37	NA	＜800

**Table 2:** Pulmonary function test FVC: forced vital capacity; FEV_1_: forced expiratory volume in 1 second; DLCO: diffusing capacity for carbon monoxide; NA: not available. DLCO reference range: This indicator's reference range requires personalized calculation based on anthropometric parameters (e.g., height/weight), and its reduction in LAM reflects cystic lung destruction with consequent gas exchange impairment.

Item	Result (2024.10)	Result (2025.03)	Reference Range
Pulmonary ventilation			
FVC (L)	4.14	4.01	NA
FVC%	127.4	124.6	≥80%
FEV_1 _(L)	3.24	3.18	NA
FEV_1_%	116.2	115.5	≥80%
FEV_1_/FVC	93.5	94.9	≥70%
Diffusing capacity			
DLCO (mmol/min/kPa/L)	71.9	67.2	NA
DLCO%	84.0	75.5	≥80%

Imaging examinations

**Figure 4:**
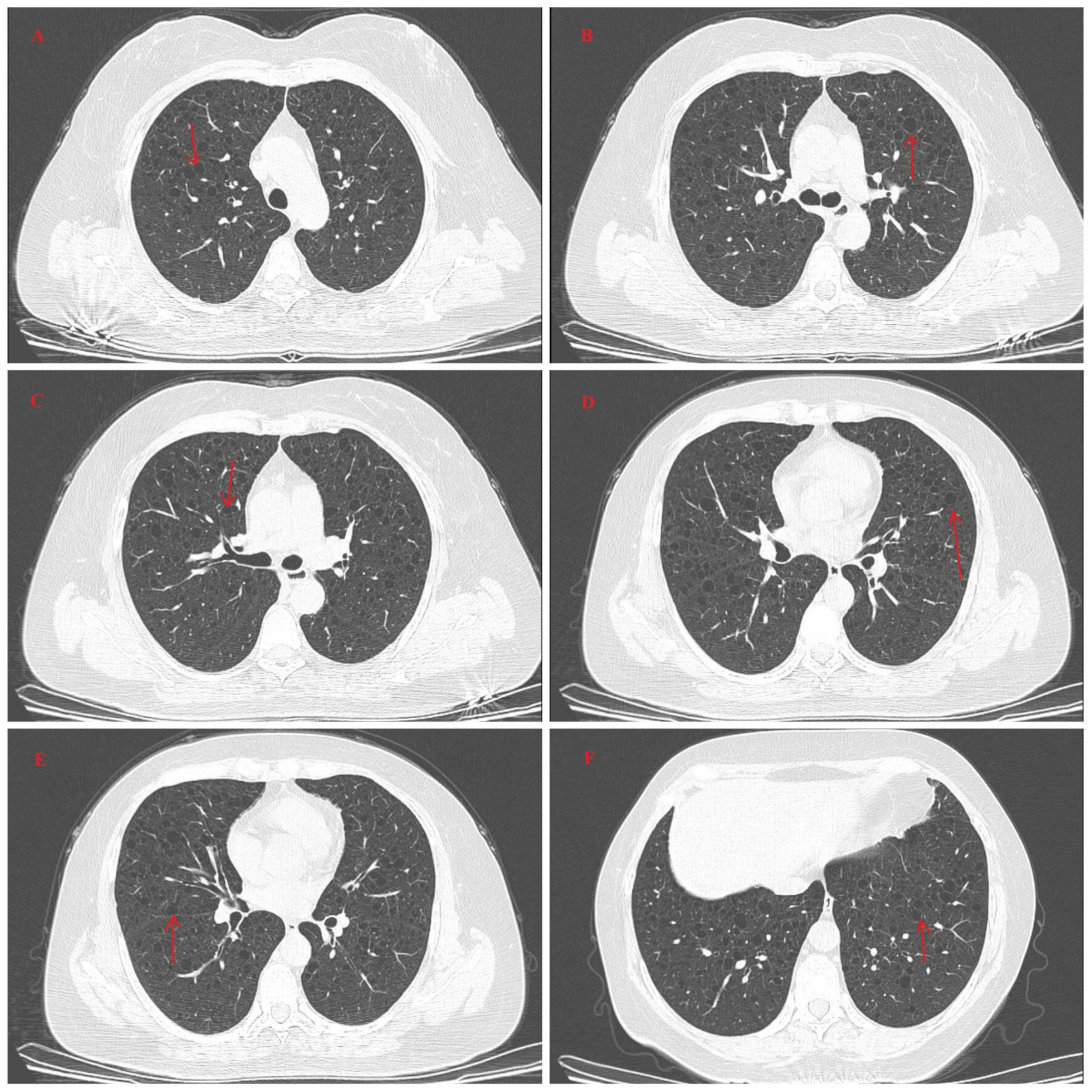
Lung CT images (October 2024) (A–F) HRCT of the lung demonstrates multiple pulmonary bullae (arrows) and diffuse emphysema in both lungs. HRCT: high-resolution computed tomography

**Figure 5:**
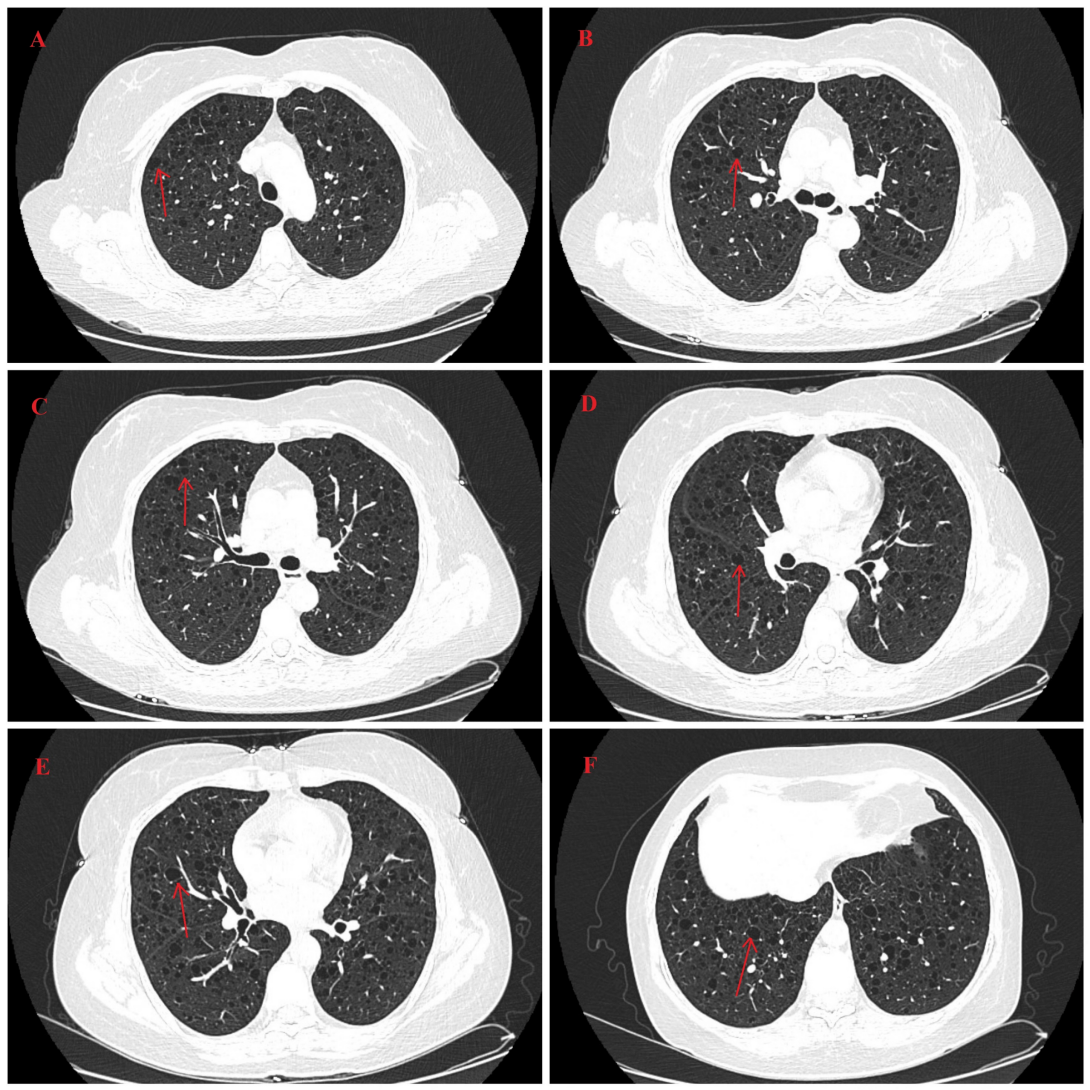
Lung CT Images (March 2025) (A–F) HRCT of the lung demonstrates diffuse emphysema and multiple cystic spaces in both lungs, suggestive of lymphocytic interstitial lung disease HRCT: high-resolution computed tomography

The lung CT in October 2024 revealed multiple cystic lucencies in both lungs. In combination with the medical history of pSS, this was initially suggestive of lymphocytic interstitial pneumonia (Figure 4). Follow-up CT revealed that emphysema and diffuse multiple cystic spaces in both lungs, so lymphoid interstitial lung disease was considered (Figure 5).

Final diagnosis, treatment, outcomes, and follow-up

The patient was ultimately diagnosed with concurrent pSS and LAM. For pSS, the patient continued mycophenolate mofetil (0.5g twice daily), hydroxychloroquine sulfate (0.1g twice daily) to regulate immunity, methylprednisolone (4mg/day) to reduce inflammation, and oral calcitriol capsules and calcium carbonate D3 tablets to prevent osteoporosis. For LAM, sirolimus was initiated at 2 mg/day and adjusted to 1 mg/day in February 2025, based on a blood concentration of 6.60 ng/ml. Pidotimod was added to enhance immunity because of long-term immunosuppression.

The patient was discharged on Day 7 with instructions for regular outpatient monitoring. 

## Discussion

This case began with a deceptively simple cough, at first misattributed to nonspecific causes or bullous lung disease, but ultimately diagnosed as a concurrence of pSS and LAM after years of uncertain diagnosis. This process emphasizes the importance of accurate diagnosis in patients with autoimmune diseases who present with atypical lung symptoms.

PSS is characterized by inflammation and destruction of the salivary and lacrimal glands [6], with pulmonary involvement including interstitial lung disease (ILD), airway disease, and lymphoproliferative disorders [7]. In this case, the patient fulfilled the 2016 ACR/EULAR classification criteria for pSS with a total score of ≥4 points: positive anti-SSA antibodies (3 points) and focal lymphocytic sialadenitis on labial gland biopsy (3 points). Although the biopsy demonstrated a small number of lymphocytic infiltrates**,** the diagnosis of pSS was maintained given the compelling clinical symptomatology. Lymphocytic interstitial pneumonia, a benign lymphoproliferative disorder in ILD, is often associated with autoimmune diseases [8]. Among them, LAM is a rare progressive cystic lung disease that mainly affects women of childbearing age [9]. In this case, the lung CT revealed diffuse bilateral pulmonary cystic lesions, with several demonstrating notably enlarged diameters. Given the absence of pathognomonic clinical features (such as recurrent pneumothorax or chylothorax) and negative histopathological/laboratory workup for LAM, the working diagnosis favored bullous emphysema - a consideration further supported by the well-documented pulmonary manifestations in pSS. This case highlights the imperative for multidisciplinary evaluation to ensure diagnostic accuracy.

The diagnostic delay reflects the challenge of recognizing LAM in pSS patients. Studies show that [10] in patients with autoimmune diseases, tracheal and large airway involvement can lead to mucosal dryness, and symptoms such as cough and dyspnea are often attributed to ILD or other airway disorders. In addition, when lung involvement occurs, lung compliance will decrease and lung parenchymal stress will increase. After traction-induced bronchiectasis, hyperlucent shadows in the lungs may also appear and can include pulmonary emphysema and pneumatocele [11]. These factors can obscure the recognition of LAM, contributing to delayed or missed diagnoses.

To address the diagnostic challenge of distinguishing LAM from Sjögren’s Syndrome-related cystic lung disease, we summarize key differential diagnostic features to avoid misdiagnosis (Table 3) [3, 12].

**Table 3:** Differential diagnosis between LAM and Sjögren’s syndrome-related cystic lung disease In this case, the lung biopsy strongly supported the diagnosis of LAM. Additionally, imaging revealed no nodules or ground-glass opacities typical of Sjögren’s syndrome-related lung disease. The collective clinical, imaging, and pathological evidence thus confirms LAM rather than Sjögren’s syndrome-related cystic lung disease. LAM: lymphangioleiomyomatosis

Item	LAM	Sjögren’s Syndrome-Related Cystic Lung Disease
Epidemiology	Predominantly affects women of childbearing age	Commonly observed in patients with pSS, with a female predominance but no strict age restriction
Clinical Features	Characterized by chylothorax, exertional dyspnea, and in advanced stages, pulmonary hypertension or respiratory failure	Manifests with sicca symptoms (dry mouth and eyes) and interstitial lung disease, with cystic lesions as occasional pulmonary feature
Imaging Findings	Reveals diffuse, thin-walled cystic airspaces (5-10 mm) in both lungs, uniformly distributed, without nodules or ground glass opacities	Demonstrates variable-sized cystic cavities with thick walls, often accompanied by nodules, ground glass opacities, or interstitial lung disease
Histopathological Features	Smooth muscle-like cells proliferate in the interstitium, bronchi, blood vessels, and lymphatic vessels, with alveolar dilatation forming cystic air spaces	Predominantly shows lymphocyte infiltration, often with lymphoid follicular hyperplasia; cystic lesions result from inflammation and fibrosis, without smooth muscle proliferation
Immunohistochemistry	HMB-45 (+, high specificity), SMA(+), ER/PR(+)	HMB-45 (-), SMA (-), ER/PR (-), CD20/CD3 (+, indicating lymphocyte proliferation)

Fortunately, the diagnosis of LAM was confirmed by the patient’s lung CT, elevated serum VEGF-D, and characteristic lung pathology. These findings are pivotal in distinguishing LAM from other cystic lung diseases, such as Birt-Hogg-Dubé syndrome and pulmonary Langerhans cell histiocytosis. Moreover, after choosing sirolimus treatment, the patient’s symptoms improved significantly, which proved that the clinical diagnosis was correct. In pSS, immune dysfunction driven by mechanisms like heightened B-cell activity and chronic lymphocyte infiltration creates a microenvironment conducive to abnormal pulmonary smooth muscle hyperplasia [13]. Research has shown [14] that functional loss mutations in tuberous sclerosis complex (TSC) 1 and 2 can be broadly detected in LAM cells, activating the mechanistic target of rapamycin (mTOR) pathway, which also plays a crucial role in B-cell hyperactivation [15]. Although a few case reports [16,17] have documented the coexistence of pSS and LAM, it remains unclear whether these conditions share common pathogenic pathways or represent a coincidental association. The current case could advance clinical vigilance for complex rare disorders and offer a springboard for future research.

Therapeutically, the patient was prescribed sirolimus, an mTOR inhibitor, which has been shown to improve lung function and slow disease progression in LAM [18,19]. For pSS, the glucocorticoid treatment was combined with immunosuppressive therapy, aligning with clinical guidelines [20]. During follow-up, the patient’s lung function and pulmonary lesions remained stable, which suggests effective disease control. Both diseases have chronic and progressive characteristics, so long-term follow-up is essential.

Later, we reviewed this case and identified several key clinical lessons. First, distinguishing LAM from emphysema on lung CT is critical: emphysema manifests as asymmetric, upper-lobe-predominant air-filled spaces with imperceptible walls due to alveolar septal destruction, whereas LAM presents as diffuse, uniform thin-walled cysts. Second, in pSS patients with refractory respiratory symptoms not fully explained by imaging, comprehensive evaluation, including lung biopsy, is warranted to rule out or diagnose rare entities like LAM. Finally, the potential interplay between mTOR activation and autoimmune dysregulation warrants further research to elucidate whether pSS predisposes to LAM or whether their coexistence is coincidental. These insights underscore the importance of integrating advanced diagnostics and translational research to improve the symptoms of patients.

## Conclusions

The coexistence of pSS and LAM in this 47-year-old woman highlights the diagnostic challenges of rare overlapping diseases. Clinicians should maintain a high suspicion of atypical pulmonary symptoms in autoimmune diseases, leveraging imaging, serology, and histopathology to promptly recognize changes and accurately diagnose. Early identification and timely treatment are critical for improving patient prognosis. Future research can further explore the potential mechanistic links between pSS and LAM.
